# Draft Genome Sequence of Phosphate-Solubilizing Bacterium *Paraburkholderia tropica* Strain P-31 Isolated from Pomegranate (*Punica granatum*) Rhizosphere

**DOI:** 10.1128/genomeA.00844-16

**Published:** 2016-08-18

**Authors:** Chandandeep Kaur, Govindan Selvakumar, Arakalgud Nanjundiah Ganeshamurthy

**Affiliations:** a Division of Soil Science and Agricultural Chemistry, ICAR—Indian Institute of Horticultural Research, Hesarghatta Lake Post, Bengaluru, India; b Jain University, Bengaluru, India

## Abstract

We report the 8.9 Mb draft genome sequence of phosphate-solubilizing bacterium *Paraburkholderia tropica* strain P-31, isolated from pomegranate (*Punica granatum*) rhizosphere. The draft genome sequence of *Paraburkholderia tropica* strain P-31 consists of 8,881,246 bp with a G+C content of 64.7%, 8,039 protein-coding genes, and 49 RNAs.

## GENOME ANNOUNCEMENT

*Burkholderia* (family *Burkholderiaceae*) is a large and ubiquitous genus within *Betaproteobacteria* (1). While some species are reported to be pathogenic to humans and animals *viz.,* the *B. cepacia* complex (Bcc), *B. pseudomallei*, and *B. mallei* (2–5), several strains are environmental in nature and possess plant growth-promotion traits. Based on phylogenetic affinity, it has been recently proposed to divide the genus into two distinct groups *viz., Burkholderia* and *Paraburkholderia.* While the former accommodates clinical isolates, the latter encompasses the plant beneficial environmental (PBE) group (6, 7). Another proposal has been made to accommodate selected members of the genera *Burkholderia* and *Paraburkholderia* in the genus *Caballeronia* (8).

*Paraburkholderia tropica* is a phosphate-solubilizing bacterial strain isolated from the rhizosphere of pomegranate (*Punica granatum*). The genomic DNA was extracted from an exponentially grown culture using ZR Fungal/Bacterial DNA MiniPrep as per manufacturer’s instructions. The genome was sequenced using a standard Illumina-HiSeq 1000 technology. A total of 20,590,078 reads were generated, amounting to 3,070,952,404 bp, and were *de novo* assembled using CLC Genomics Workbench version 7.5.1 (CLC bio, Aarhus, Denmark) into 148 contigs, with a total length of 8,905,186 bp and mean coverage of 100×. The assembly has a *N*_50_ of 89,003 bp and average contig length of 60,170 bp, with a mean G+C content of 64.7%. The functional annotation was carried out by RAST (Rapid Annotation using Subsystem Technology), tRNA was predicted by ARAGORN (9), and rRNA genes by RNAmmer 1.2 (10). The genome contains a total of 8,039 coding sequences (CDSs) and 49 RNAs were predicted.

Whole-genome annotation with the RAST server shows that strain P-31 possesses multiple genes that play an important role in phosphate solubilization; these include the PQQ dependent glucose dehydrogenase (GDH) responsible for the periplasmic oxidation of glucose to gluconic acid, citrate synthase and lactate dehydrogenase responsible for production of citric and lactic acid, respectively (11). Genes encoding a phosphoenolpyruvate carboxylase (Pepc) that increases the supply of oxaloacetate, a crucial anabolic precursor and an intermediate in biosynthesis of organic acids implicated in phosphate (P) solubilization (12) are also encoded. The genes responsible for phosphate metabolism present in the genome include the ABC transporter complex, i.e., Pst ABCS responsible for inorganic phosphate (Pi) uptake under Pi starvation conditions, the Pho regulon which includes genes for alkaline phosphatase (AP) activity, the PhoB-PhoR proteins, PstABCS, and PhoU proteins (13, 14). These genes are co-regulated by extracellular phosphate and are involved in phosphorous assimilation. Enzymes like exopolyphosphatase (Ppx) and polyphosphate kinase (Ppk), which catalyze the hydrolysis of inorganic polyphosphate P, to release orthophosphate are also encoded in the genome. Apart from genes responsible for phosphate solubilization and metabolism, genes encoding plant growth-promotion traits such as indole oxidoreductase subunit (IorA), indole-3-glycerol phosphate synthase (TrpD), and tryptophan synthase (TrpEa, TrpEb) were also detected in the genome of *Paraburkholderia tropica*.

### Accession number(s).

This whole-genome shotgun project has been deposited at DDBJ/ENA/GenBank under the accession no. LXGI00000000. The version described in this paper is version LXGI01000000.
